# Influence of presence/absence of thyroid gland on the cutoff value for thyroglobulin in lymph-node aspiration to detect metastatic papillary thyroid carcinoma

**DOI:** 10.1186/s12885-017-3296-3

**Published:** 2017-04-28

**Authors:** Huan Zhao, Yong Wang, Min-jie Wang, Zhi-hui Zhang, Hai-rui Wang, Bing Zhang, Hui-qin Guo

**Affiliations:** 1 Department of Pathology, National Cancer Center/Cancer Hospital, Chinese Academy of Medical Sciences and Peking Union Medical College, 17 Nanli Panjiayuan Lane, Chaoyang District, Beijing, 100021 People’s Republic of China; 2 Department of Diagnostic Radiology, National Cancer Center/Cancer Hospital, Chinese Academy of Medical Sciences and Peking Union Medical College, Beijing, People’s Republic of China; 3 Department of Clinical Library, National Cancer Center/Cancer Hospital, Chinese Academy of Medical Sciences and Peking Union Medical College, Beijing, People’s Republic of China; 4 Department of Cancer Epidemiology, National Cancer Center/Cancer Hospital, Chinese Academy of Medical Sciences and Peking Union Medical College, Beijing, People’s Republic of China; 5 Department of Head and Neck Surgery, Peking University Cancer Hospital, Beijing, China

**Keywords:** Thyroglobulin, Fine-needle aspirate, Cutoff, Papillary thyroid carcinoma, Lymph node

## Abstract

**Background:**

Thyroglobulin measurement with fine-needle aspiration (Tg-FNA) is a sensitive method for detecting metastatic papillary thyroid carcinoma (PTC). However, the diagnostic threshold is not well established and the influence of the thyroid gland on the cutoff value is also controversial. In this study, patients were classified into two groups according to the presence or absence of thyroid tissue, to determine an appropriate cutoff value for clinical practice.

**Methods:**

Patients with a history of thyroid nodules or surgery for PTC and with enlarged cervical lymph nodes on an FNA examination were enrolled for Tg-FNA detection.

**Results:**

One hundred ninety-six lymph nodes (189 patients) were included: 100 from preoperative patients, 49 from patients treated with partial thyroid ablation, and 47 from patients with total thyroid ablation. In 149 lymph nodes from patient with thyroids, the cutoff value for Tg-FNA was 55.99 ng/mL (sensitivity, 95.1%; specificity, 100%), whereas in 47 lymph nodes from patients without a thyroid, it was 9.71 ng/mL (sensitivity, 96.7%; specificity, 100%). Thus, the cutoff value for Tg-FNA was higher in patients with thyroids than in patients without thyroids.

**Conclusions:**

The cutoff value for Tg-FNA is influenced by residual thyroid tissue, and a higher cutoff value is recommended for patients with thyroids than for patients without thyroids.

**Electronic supplementary material:**

The online version of this article (doi:10.1186/s12885-017-3296-3) contains supplementary material, which is available to authorized users.

## Background

In the last three decades, thyroid carcinoma has become the most rapidly increasing disease throughout the world, including on the Chinese mainland [1]. Papillary thyroid carcinoma (PTC) accounts for the majority of all thyroid cancers. Lymph-node metastasis is a frequent finding at the onset or during the follow-up of PTC, and ultrasonography and fine-needle aspiration cytology (FNAC) are the most important modalities for the evaluation of lymph-node metastasis. Ultrasonography is highly sensitive in detecting cervical metastases, but its specificity is low [2]. Although FNAC is highly specific, its false-negative rate can be as high as 8.6%–24% because of sampling error [3, 4].

Thyroglobulin measurement with fine-needle aspiration (Tg-FNA) was initially proposed in 1992 by Pacini et al. for the detection of neck lymph-node metastasis in patients with PTC [5]. Several studies have reported that Tg-FNA is more sensitive than FNAC in detecting metastasis and that the sensitivity of FNAC is increased when combined with Tg-FNA [6–15]. However, the diagnostic threshold has not been well established. Apart from differences in the sample treatments and Tg assays, the Tg-FNA cutoff may differ in the presence or absence of the thyroid gland because thyroglobulin can be detected in the FNA washout fluid from nonmetastatic lymph nodes in the presence of a thyroid gland [13]. However, few studies have investigated the different cutoff values for Tg-FNA measurements in athyrotic patients and patients with thyroid glands [15–17].

We separated patients into two groups according to the presence or absence of the thyroid gland. One group contained patients with a thyroid, including patients awaiting surgery or after partial thyroid resection. The other contained patients who had undergone total thyroid ablation (followed or not by ^131^I ablative therapy). We analyzed the difference in the cutoff values for these two groups and evaluated the influence of the presence/absence of the thyroid gland on the cutoff value.

## Methods

### Case selection

The specimens were collected consecutively from the National Cancer Center/Cancer Hospital, Chinese Academy of Medical Sciences, between September 2012 and June 2015. The patients included in the study were selected on the basis of the following criteria: [1] enlarged cervical lymph nodes and referred to our institution by their physicians for an FNA examination; and [2] a history of thyroid nodules or surgery for PTC. In total, 189 patients and 196 lymph nodes were evaluated. The study protocol was reviewed and approved by the Ethics Committee of the National Cancer Center/Cancer Hospital. All patients gave their informed consent.

### FNA cytology

Palpable lesions were aspirated by cytopathologists, and nonpalpable lesions were aspirated by experienced radiologists under real-time ultrasound guidance. FNA was performed with a 22-gauge needle attached to a 10-mL syringe, without the aid of a syringe holder. If there was enough aspirated fluid, several drops of it were first added to 0.5 mL of normal saline solution for the Tg measurement. The residual aspirate in the needle was then rinsed in CytoLyt (Hologic, Marlborough, MA, USA) to prepare a ThinPrep (Hologic) slide. If there was little aspirate, it was only rinsed in CytoLyt (Hologic, Marlborough, MA, USA) to prepare a ThinPrep (Hologic) slide. Another aspiration was performed and the material of the second aspirate was added to 0.5 mL of normal saline solution for Tg measurement. The cytology slides were fixed in alcohol and stained with Papanicolaou stain. They were then interpreted by cytologists with experience ranging from 5 to 18 years. The cytological diagnosis was categorized as benign, atypical, suspected PTC/malignant, or PTC/malignant. All cases, except those diagnosed cytologically as benign, were reviewed by these cytopathologists in daily conferences.

### Measurement of Tg-FNA, serum Tg, and TgAb

The FNA wash-out specimens were stored at −20 °C and transferred to the clinical laboratory within 1 month for the analysis of Tg. Serum Tg was only measured in patients on the advice of their physicians. The Tg concentrations in both the serum and FNA wash-out were measured with an automated electrochemiluminescence immunoassay (Cobas e 601, Roche Diagnostics, Mannheim, Germany). The minimum detectable Tg concentration was 0.04 ng/mL. The maximum Tg concentration detectable in our laboratory was 500 ng/mL. If the Tg level of the sample was above the maximum value (500 ng/mL), the sample was diluted and examined again. According to previous reports, the cutoff value was far lower than the maximum value [5–14]. Therefore, we decided to perform no further examination of the samples with maximum values, and recorded these values as 500+ ng/mL. Serum TgAb was also measured with an automated electrochemiluminescence immunoassay (Cobas e 601, Roche Diagnostics, Mannheim, Germany) with a functional sensitivity of 10 IU/mL.

### Data analysis and statistical analysis

The final positive diagnoses were based on histological confirmation of metastatic PTC or a cytological diagnosis of PTC. The final negative diagnoses were made based on malignancy-free lymph nodes according to cytology and negative follow-up imaging for at least 6 months, or histologically or cytologically confirmed lymph-node metastases from extrathyroidal malignancies.

The cytological results were grouped into two categories according to the cytology reports. Cases with reports documenting PTC and those documenting suspected PTC were considered positive. Negative diagnoses were assigned to (1) cases with reports where ‘atypical’ was mentioned but ‘PTC’ was not; (2) cases of lymph nodes with reactive hyperplasia; and (3) cases with reports of extrathyroidal malignancies.

‘TgAb positive’ was defined as serum TgAb > 60 IU/mL and ‘TgAb negative’ as serum TgAb ≤60 IU/mL, according to a previous report [18].

A receiver operating characteristic (ROC) curve analysis was conducted to determine the most appropriate threshold value for Tg-FNA, with the areas under the ROC curves (AUCs) and confidence intervals (CIs) assessed with MedCalc version 14.10.2. Comparisons of significant differences between groups were made with the χ^2^ test. Statistical analyses were performed with SPSS 12.0 (SPSS Inc., Chicago, IL, USA), and *P* < 0.05 was considered statistically significant.

## Results

### Characteristics of patients and lymph nodes

In this study, 196 lymph nodes (189 patients) were enrolled: 100 (51.0%) from preoperative patients, 49 (25.0%) from patients treated with partial thyroid ablation, and 47 (24.0%) from patients with total thyroid ablation. Thirty-seven of the 91 patients (96 lymph nodes) who underwent surgery did so in our hospital, and the details of their operations are shown in Additional file 1: Table S1. (Because the other 54 patients did not undergo surgery in our hospital, we cannot present the details of their operations; the presence/absence of thyroid was evaluated with ultrasound at our hospital). Of the lymph nodes from patients who had undergone total thyroid ablation, 24 had been treated with ^131^I ablation therapy.

In the final diagnosis, 111 nodes were positive for metastatic PTC (one special case was diagnosed as metastatic thyroid carcinoma, in which the tumor was mainly composed by squamous carcinoma, with a small amount of papillary carcinoma), and the remaining 85 were negative (50 lymphadenitis and 35 metastases from extrathyroidal malignancies). In patients with metastatic PTC, 17 (15.3%) lymph nodes had a maximum diameter of ≤1 cm and 49 (44.1%) were cystic (Table 1).

**Table 1:** Clinical characteristics of 196 lymph nodes

	Metastatic PTC (*n* = 111)	Lymphadenitis (*n* = 50)	Metastases from extrathyroidal malignancies (*n* = 35)
Patient’s thyroid conditions			
No-surgery	48 (43.2%)	19 (38.0%)	33 (94.3%)
Partial ablation	33 (29.7%)	14 (28.0%)	2 (5.7%)
Total ablation	30 (27.0%)	17 (34.0%)	0 (0%)
Size of lymph node			
≤ 1 cm	17 (15.3%)	8 (16.0%)	0 (0.0%)
> 1 cm	94 (84.7%)	42 (84.0%)	35 (100.0%)
Characteristics Of lymph node			
cystic	49 (44.1%)	3 (6.0%)	16 (45.7%)
solid	62 (55.9%)	47 (94.0%)	19 (54.3%)

### Optimal cutoff value for Tg-FNA measurement

The values for Tg-FNA ranged from 0.642 ng/mL to 500+ ng/mL (median 500+ ng/mL) in metastatic PTC lymph nodes; from 0.04 ng/mL to 13.06 ng/mL (median 1.525 ng/mL) in lymphadenitis; and from 0.1 to 52.2 ng/mL in metastases from extrathyroidal malignancies (median 1.24 ng/mL). We used ROC curves to evaluate the diagnostic capacity of Tg-FNA to detect metastatic PTC. The AUCs were 0.999 (95% CI, 0.997–1.000) in patients with thyroids and 0.984 (95% CI, 0.952–1.000) in patients without thyroids. The cutoff value for Tg-FNA was higher in patients with thyroids than in patients without thyroids. In the 149 lymph nodes from patients with thyroids, the cutoff value for FNA-Tg was 55.99 ng/mL (sensitivity, 95.1%; specificity, 100%), whereas in the 47 lymph nodes from patients without thyroids, it was 9.71 ng/mL (sensitivity, 96.7%; specificity, 100%).

### Cutoff value at Tg-FNA/serum-Tg ratio > 1.0 for Tg-FNA measurement

The serum Tg levels were evaluated in 68 patients before the FNA examination. The periods between the serum Tg examination and the FNA examination ranged from 0 to 60 days (median 7.5 days). In 68 patients with recorded serum Tg levels, 59 had metastatic PTC and nine had reactive lymph nodes on the final diagnosis. When the cutoff value of the Tg-FNA/serum-Tg ratio was >1.0, the sensitivity and specificity of the Tg-FNA measurement were 89.8% and 77.8%, respectively.

### Diagnostic ability of FNAC

FNAC correctly diagnosed 99 of 111 metastatic PTCs, but failed to diagnose 12. Among the 12 cytology-missed lymph nodes, nine were cystic and three were solid. The maximum diameter of two of the solid nodes was <1 cm. FNAC correctly diagnosed all 50 nodes from patients with lymphadenitis, whereas in 35 lymph nodes from patients with metastases from extrathyroidal malignancies, FNAC missed one case of cystic squamous carcinoma and correctly diagnosed the others (Table 2). When identifying metastatic PTC, the sensitivity, specificity, and diagnostic accuracy of FNAC were 89.2% (99/111), 100% (85/85), and 93.9% (184/196), respectively.

**Table 2:** Fine-needle aspiration cytology vs. final diagnosis

Cytology diagnosis	Final diagnosis		
	Metastatic PTC (*n* = 111)	Lymphadenitis (*n* = 50)	Metastases from extrathyroidal malignancies (*n* = 35)
Benign	7	49	1
Atypical	5	1	0
Suspicious PTC/Malignance	22	0	6
PTC/Malignant	77	0	28

### Diagnostic performance of FNAC combined with Tg-FNA measurement

The diagnostic strategy using FNAC alone had a sensitivity of 89.2% and a specificity of 100% in determining metastatic PTC, whereas a higher sensitivity (99.1%, *P* = 0.002) and similar specificity (100%, *P* = 1.000) were obtained with a diagnostic strategy that combined Tg-FNA and FNAC. With this strategy, the cutoff value was 55.99 ng/mL for patients with thyroids and 9.71 ng/mL for patients without thyroids (Table 3). Twelve lymph nodes with metastatic PTC were misdiagnosed by FNAC. Among these 12 cases, 11 were detected by Tg-FNA measurements. The specific Tg-FNA levels in the 12 cases missed by FNAC are listed in Table 4.

**Table 3:** Evaluation of metastatic PTC according to the diagnostic modality

Modalities	SN	SP	PPV	NPV	AC
FNAC	89.2% (99/111)	100%(85/85)	100%(99/99)	87.6%(95/107)	93.9%(184/196)
Tg-FNA^a^	95.5%(106/111)	100%(85/85)	100%(106/106)	94.4%(85/90)	97.4%(191/196)
FNAC + Tg-FNA^b^	99.1%(110/111)	100%(85/85)	100%(110/110)	98.8%(85/86)	99.5%(195/196)

*Abbreviations*: *SN* sensitivity; *SP* specificity; *PPV* positive predictive value; *NPV* negative predictive value; *AC* accuracy

^a^Optimal cutoff value for Tg-FNA is 55.99 ng/mL for patients with thyroids and 9.71 ng/mL for patients without thyroids

^b^Positive result was determined if the positive criteria were met in either criteria

**Table 4:** Specific Tg-FNA levels of 12 lymph nodes missed by FNAC

No.	Thyroid condition	Lymph-node characteristics	Cytological diagnosis	Tg-FNA (ng/mL)	Final diagnosis
1	Partial ablation	Cystic	Atypical cells	>500	Metastatic PTC
2	Partial ablation	Cystic	Atypical cells	>500	Metastatic PTC
3	Partial ablation	Cystic	Atypical cells	>500	Metastatic PTC
4	Before surgery	Cystic	Atypical cells	259.6	Metastatic PTC
5	Before surgery	Cystic	Histocytes	>500	Metastatic PTC
6	Before surgery	Cystic	Histocytes	>500	Metastatic PTC
7	Total ablation	Cystic	Histocytes	>500	Metastatic PTC
8	Before surgery	Cystic	Histocytes	166.5	Metastatic PTC
9	Total ablation	Cystic	Histocytes	0.642	Metastatic carcinoma (squamous carcinoma + a small amount of PTC)
10	Total ablation	Solid	Lymphocytes	12.80	Metastatic PTC
11	Total ablation	Solid	Lymphocytes	20.12	Metastatic PTC
12	Partial ablation	Solid	Atypical cells	447.40	Metastatic PTC

Optimal cut-off value for Tg-FNA is 55.99 ng/mL for patients with thyroids and 9.71 ng/mL for patients without thyroids

### Influence of serum TgAb on the diagnostic efficacy of Tg-FNA

The influence of serum TgAb on the diagnostic performance of Tg-FNA is shown in Table 5. In the TgAb-positive group, Tg-FNA had a sensitivity of 92.3% in identifying metastatic PTC. However, in the TgAb-negative group, the sensitivity of Tg-FNA was 95.7%. Therefore, the diagnostic capacity of Tg-FNA was similar in the two different groups (*P* = 0.628).

**Table 5:** Influence of presence/absence of serum TgAb on the diagnostic capacity of Tg-FNA

	Tg-FNA+^a^	Tg-FNA−
Serum TgAb+^b^		
Metastatic PTC (*n* = 13)	12	1
Lymphadenitis (*n* = 0)	0	0
Serum TgAb−		
Metastatic PTC (*n* = 46)	44	2
Lymphadenitis (*n* = 9)	0	9

^a^Tg-FNA positive (Tg-FNA+) was defined as a Tg-FNA level ≥ 55.99 ng/mL for patients with thyroids and 9.71 ng/mL for patients without thyroids

^b^TgAb positive (TgAb+) was defined as a serum TgAb >60 IU/mL

### Cases managed with FNAC combined with Tg-FNA measurement

In the last stage of the study, a Tg-FNA measurement was made as a clinical assessment. The decision for surgery was based not only to the FNA results but also on the Tg-FNA level. Additional file 2: Table S2 shows all the patients managed with FNAC combined with Tg-FNA measurement. Among the nine patients, the clinical management of three was altered based on their Tg-FNA measurements. Two patients (case 5 and case 9), with negative cytology results, showed significantly elevated Tg-FNA levels, and were therefore easily diagnosed with metastatic PTC. Another patient (case 3) with negative cytology results showed slightly elevated Tg-FNA (12.8 ng/mL). In this situation, it was hard for surgeons to make a decision. Because this patient had a history of total thyroid ablation and a serum Tg level of <0.04 ng/mL, the surgeon believed that 12.8 ng/mL was abnormal for this patient and suggested that the patient be managed with surgery. This lymph node was ultimately found to be metastatic PTC during surgery.

## Discussion

Tg-FNA measurement is a useful tool for detecting metastases from PTC, with high sensitivity and specificity, in both the preoperative and postoperative context. Although this procedure is now recommended by the American and European Thyroid Association guidelines [19, 20], the cutoff value for Tg-FNA is still controversial [6–17]. In previous reports, the functional sensitivity of Tg measurements was the most commonly used threshold values [7, 10–12]. However, this threshold value suffers from several limitations. Tg-FNA may be higher if the patient has a remnant thyroid gland. The aspirate may be contaminated with blood containing high levels of Tg, or the Tg secreted by the remnant thyroid may be transported to the regional lymph node, like the dye used to map the sentinel lymph node during the operation. In patients awaiting surgery or even in postoperative patients with partial thyroid ablation, Tg secretion may not be suppressed, which can confuse the diagnosis. However, few studies have investigated the difference in the cutoff values for athyrotic patients and patients with intact thyroid tissue [15–17].

In the past, the surgical and radioiodine ablation of cancerous and all noncancerous thyroid cells is the most common management regimen for PTC in Western countries. Therefore, the influence of an intact thyroid can be ignored in postoperative patients in the West. However, in the Chinese population, various surgical procedures are used for PTC, including total ablation, lobectomy, lobe and isthmus resection, and affected lobe and isthmus resection plus opposite-side subtotal lobectomy [21]. In this study, 96 lymph nodes were from patients after thyroid surgery. Of these, 47 were from collected patients who had undergone total thyroid ablation, and 49 were from patients after partial resection. Therefore, the influence of the remnant thyroid gland on the cutoff value for Tg-FNA must be considered, even in postoperative patients in the Chinese population. Recently, the 2015 American Thyroid Association guidelines recommended that, for low-risk papillary and follicular carcinomas between 1 and 4 cm in size, thyroid lobectomy alone may be sufficient as an initial treatment [19]. Therefore, we believe that the influence of the remnant thyroid gland on the cutoff value for Tg-FNA is a common problem throughout the world.

We separated the patients into two groups, with or without thyroid, to evaluate the influence of intact thyroid tissue. The group with thyroids was composed of patients awaiting surgery and patients after partial thyroid resection. The other group, without thyroids, was composed of patients after total thyroid ablation (with or without subsequent ^131^ I ablative therapy). The optimal cutoff value we recommend for patients with thyroids is 55.99 ng/mL (sensitivity, 95.1%; specificity, 100%), whereas that for patients without thyroids is 9.71 ng/mL (sensitivity, 96.7%; specificity, 100%). In clinical practice, PTC generally behaves indolently. Therefore, we consider that the specificity of Tg-FNA is highly significant, in reducing false positive results as far as possible and avoiding unnecessary surgery. Therefore, we selected a cutoff value with high specificity as the optimal cutoff value.

Our results support the hypothesis that remnant thyroid increases the Tg-FNA level. For patients with thyroids, the high cutoff level for Tg-FNA can be attributed to the remnant thyroid, as is readily understandable. An elevated cutoff value of 32.04 ng/mL was also recommended for preoperative patients in the study by Pak et al. [17]. However, the high levels of Tg-FNA in the group of patients who had undergone total thyroid ablation are more difficult to understand. In Western countries, surgical and radioiodine ablation of cancerous and all noncancerous thyroid cells is the most common management regimen for PTC. However, in our study, less than half the patients had undergone total thyroid ablation had ^131^I ablative therapy. Residual thyroid tissue without ^131^I ablation may have also contributed to the elevated level of Tg-FNA. Therefore, the influence of remnant thyroid gland must be considered in all FNA patients, even in those in the Chinese population who have undergone total thyroid ablation.

Actually, the influence of residual thyroid tissue on Tg-FNA levels has also been noted in reports from Western countries. In a series of reports from American and Australia, although the functional sensitivity of Tg-FNA was recommended as the cutoff value, the specificity of Tg-FNA was as low as 50%–87.5% [10–12]. This means that 12.5%–50% of reactive lymph nodes in postoperative patients in those studies had elevated Tg-FNA levels. An elevated threshold can reduce the false positive rate and avoid unnecessary surgery, which is important for thyroid carcinoma patients who have already undergone many rounds of surgery.

The serum Tg level has also been used as the threshold value in previous studies [22, 23]. If the Tg-FNA/serum-Tg ratio is >1.0, it is assumed that Tg-FNA is positive. However, using the Tg-FNA/serum-Tg ratio as the detection threshold may be dubious in the following situations. First, when patients who have not undergone a serum-Tg evaluation, we cannot compare the Tg-FNA levels with the serum levels. Second, in patients where blood is not sampled at the same time for serum-Tg and Tg-FNA, but are only sampled within a certain time period, there can be hormonally induced variations in the serum-Tg within this period, which may skew the data. In our study, serum Tg levels were only evaluated in 68 patients, so patients with serum Tg data accounted for 36.0% of all the patients. The time intervals ranged from 0 to 60 days (median 7.5 days) from the time of serum Tg sampling to the time of FNA. Using a cutoff value of FNA-T/serum-Tg ratio > 1.0, the sensitivity and specificity of the Tg-FNA measurement were 89.8% and 77.8%, respectively. Both the sensitivity and specificity based on a cutoff value of FNA-T/serum-Tg ratio > 1.0 were lower than those based on the optimal cutoff value for Tg-FNA. This result is as same as that of Moon [15].

Consistent with previous reports, the false negative diagnoses made with FNAC in our study were mainly attributable to cystic changes in the lymph nodes. Twelve metastatic lymph nodes were missed by FNAC. Among these, nine were cystic and two were small lymph nodes, with maximum diameters of ≤1 cm. The inefficiency of FNAC has also been attributed to small lymph nodes in a previous study [24]. The sensitivity of detecting lymph-node metastasis was greatly increased by combining Tg-FNA and cytology, and 11 of 12 metastatic lymph nodes with negative cytology were correctly identified. The one case of metastatic PTC missed by Tg-FNA was diagnosed histologically as metastatic thyroid carcinoma, which was mainly composed of a squamous carcinoma and a small amount of papillary carcinoma. It has been reported that undetectable Tg not only occurs in metastatic lymph nodes from anaplastic or undifferentiated PTCs, but also in metastatic lymph nodes from recurrent PTCs [16]. Of all differentiated thyroid carcinomas, 2%–5% will lose their differentiated features [25], and the case reported in this study falls into this category. This patient had a history of thyroidectomy for PTC, which had recurred three times. Therefore, we recommend, as discussed previously, the use of combined cytology and Tg-FNA, rather than either technique alone, to detect metastasis from any histological type of thyroid cancer [16].

It is also unclear whether serum TgAb can influence the Tg-FNA level. Studies by Baskin and Boi et al. have shown that serum TgAb did not interfere with the Tg-FNA measurements when diagnosing metastatic PTC [2, 16]. However it has recently been suggested in the study by Shin et al. that the presence of serum TgAb can reduce the diagnostic performances of Tg-FNA [26]. Our data showed no difference in the diagnostic capacity of Tg-FNA in two groups, with and without TgAb. However, data on serum TgAb levels were only available for 36.0% (68/189) of the patients. These results require validation with a larger sample.

## Conclusions

The results of the present study demonstrate that Tg measurement with fine-needle aspiration is a useful ancillary test that improves the detection of metastatic PTC. The cutoff value for Tg-FNA is influenced by residual thyroid tissue and a higher cutoff value is recommended for patients with thyroids than for patients without thyroids. This is our first study of the cutoff values for Tg-FNA and a further large-scale study is required to validate these results.

## Additional files

Additional file 1: Table S1. Detailed surgical methods used in patients treated at our hospital. There are thirty-seven patients who underwent surgery in our hospital. The details of their operations are shown in Additional file 1: Table S1. (DOC 29 kb)

Additional file 2: Table S2. Cases managed by FNA cytology combined with Tg-FNA measurement. There are 9 patients their decisions for surgery were based not only to the FNA results but also on the Tg-FNA level. The detailed data of each patient were shown in Additional file 2: Table S2. (DOC 38 kb)

## Abbreviations

AUC: area under the curve
CI: confidence interval
FNA: fine-needle aspiration
FNAC: fine-needle aspiration cytology
PTC: papillary thyroid carcinoma
ROC: receiver operating characteristic
Tg: thyroglobulin
TgAb: anti-thyroglobulin antibody
Tg-FNA: thyroglobulin measurement with fine-needle aspiration
