# Supplementary material for: Influence of presence/absence of thyroid gland on the cutoff value for thyroglobulin in lymph-node aspiration to detect metastatic papillary thyroid carcinoma
Source: BMC Cancer. 2017 Apr 28;17:296. doi: 10.1186/s12885-017-3296-3 (PMC5410021; doi:10.1186/s12885-017-3296-3)
Supplement: Supplementary file 1 — Detailed surgical methods used in patients treated at our hospital. There are thirty-seven patients who underwent surgery in our hospital. The details of their operations are shown in Additional file 1: Table S1. (DOC 29 kb) [file 12885_2017_3296_MOESM1_ESM.doc]

Table S1 Detailed surgical methods used in patients treated at our hospital

| Operation methods | n |
| --- | --- |
| Total thyroidectomy + central neck dissections | 4 |
| Total thyroidectomy + whole neck dissections | 17 |
| Total thyroidectomy + lateral neck dissections | 1 |
| Partial thyroidectomy + central neck dissections | 4 |
| Partial thyroidectomy + whole neck dissections | 6 |
| Partial thyroidectomy + lateral neck dissections | 1 |
| Partial thyroidectomy without neck dissections | 4 |
| Total | 37 |
