# Supplementary material for: Influence of presence/absence of thyroid gland on the cutoff value for thyroglobulin in lymph-node aspiration to detect metastatic papillary thyroid carcinoma
Source: BMC Cancer. 2017 Apr 28;17:296. doi: 10.1186/s12885-017-3296-3 (PMC5410021; doi:10.1186/s12885-017-3296-3)
Supplement: Supplementary file 2 — Cases managed by FNA cytology combined with Tg-FNA measurement. There are 9 patients their decisions for surgery were based not only to the FNA results but also on the Tg-FNA level. The detailed data of each patient were shown in Additional file 2: Table S2. (DOC 38 kb) [file 12885_2017_3296_MOESM2_ESM.doc]

Table S2 Cases managed by FNA cytology combined with Tg-FNA measurement

| Case No. | Thyroid condition | Serum Tg  (ng/mL) | Serum ATG  (IU/mL) | Tg-FNA  (ng/mL) | Lymph node  characteristics | Cytology | Management | Final diagnosis |
| --- | --- | --- | --- | --- | --- | --- | --- | --- |
| 1 | Total ablation | ＜0.04 | 11.26 | ＜0.04 | Solid, 〉1cm | Lymphcytes | No-surgery | Negative |
| 2 | Total ablation | 4.47 | 472.4 | 295.1 | Cystic, ＞1cm | PTC | Surgery | Metastatic PTC |
| 3 | Total ablation | ＜0.04 | 615.4 | 12.8 | Solid, ≤1cm | Lymphcytes | Surgery | Metastatic PTC |
| 4 | Total ablation | ＜0.04 | 432.7 | ＞500 | Solid, ≤1cm | PTC | Surgery | Metastatic PTC |
| 5 | Before surgery | 48.05 | 13.28 | 259.6 | Cystic, ＞1cm | Atypical cells | Surgery | Metastatic PTC |
| 6 | Partial ablation | 5.39 | 10.68 | ＞500 | Cystic, ＞1cm | PTC | Surgery | Metastatic PTC |
| 7 | Partial ablation | 1.76 | 385.5 | 59.75 | Solid, ＞1cm | PTC | Surgery | Metastatic PTC |
| 8 | Partial ablation | 1.23 | ＜10.00 | 0.04 | Solid, ＞1cm | Lymphcytes | No-surgery | Negative |
| 9 | Before surgery | 30.9 | ＜10.00 | 166.5 | Cystic, ＞1cm | Histocytes | Surgery | Metastatic PTC |
